# RASE: Modeling cumulative disadvantage due to marginalized group status in academia

**DOI:** 10.1371/journal.pone.0260567

**Published:** 2021-12-16

**Authors:** Sarah Shandera, Jes L. Matsick, David R. Hunter, Louis Leblond

**Affiliations:** 1 Department of Physics, The Pennsylvania State University, University Park, PA, United States of America; 2 Department of Psychology, The Pennsylvania State University, University Park, PA, United States of America; 3 Department of Women’s, Gender, and Sexuality Studies, The Pennsylvania State University, University Park, PA, United States of America; 4 Department of Statistics, The Pennsylvania State University, University Park, PA, United States of America; Indiana University Bloomington, UNITED STATES

## Abstract

We propose a framework of Resources, Achievement, Status, and Events (RASE) that allows the many disparate but well-documented phenomena affecting underrepresented groups in STEM to be assembled into a story of career trajectories, illuminating the possible cumulative impact of many small inequities. Our framework contains a three-component deterministic cycle of (1) production of Achievements from Resources, (2) updated community Status due to Achievements, and (3) accrual of additional Resources based on community Status. A fourth component, stochastic Events, can influence an individual’s level of Resources or Achievements at each time step of the cycle. We build a specific mathematical model within the RASE framework and use it to investigate the impact of accumulated disadvantages from multiple compounding variables. We demonstrate that the model can reproduce data of observed disparities in academia. Finally, we use a publicly available visualization and networking tool to provide a sandbox for exploring career outcomes within the model. The modeling exercise, results, and visualization tool may be useful in the context of training STEM faculty to recognize and reduce effects of bias.

## Introduction

Diversity and equity researchers have long posited that “molehills can become mountains” [1] or “a ton of feathers still weighs a ton” [2] to explain how seemingly small, subtle, and short-term inequities contribute to long-term disparities in success. This type of macroscopic theorizing is used to elucidate observed disparities in academia [1–5]. For example, in terms of gender disparities, men typically outnumber women in full-time university faculty positions and women in the academy earn less than their male colleagues [6, 7]. Science, technology, engineering, and mathematics (STEM) in particular have persistently maintained demographic group makeup unrepresentative of the general population, despite evidence that there is more within-group variability than between-group differences in STEM-relevant skills [8–11].

A robust body of research in the social sciences addresses why disparities in representation and academic success persist in STEM. Although many isolated inequities in the experiences of marginalized groups may seem small, the accretion of these ostensibly minor events can lead to significant disparities over the course of an academic career [1, 12, 13]. The probability of accumulation of disadvantage is especially striking for women of color [14] and others who have multiple marginalized identities. For studies examining potential bias and disadvantage in academia, it is important for researchers to investigate contributing factors of disparate outcomes narrowly and in as well-controlled a way as possible. For example, some research has examined the inequitable distribution of resources differently provided to underrepresented and majority group faculty (e.g., in awarded grant funding; [15–18]), whereas other research has focused on disparities in achievements (e.g., evaluation of expertise and credentials; [3, 19–22]).

To understand how these individually-documented effects may compound over the course of a career we: (1) introduce a framework for understanding disadvantage using four elements—Resources, Achievements, Status, and Events (RASE), (2) situate those elements in evidence of social disparities in academia, and (3) build and test the utility of a specific mathematical model within RASE for explaining inequities in academic trajectories. While the framework of four elements is broad, the mathematical relationship between them can be varied. We use “model” to refer to the functional form used to define and relate the variables. The framework organizes a large literature into a few variables and allows competing mathematical models for the relationships between the variables to be explored. We present an agent-based simulation of the model [23] in NetLogo [24] and use it to generate accessible visualizations of the cumulative effect of small inequities on academic careers. We demonstrate that real-world data on inequities in academia can be reproduced by adjusting model parameters that capture the level of disadvantage experienced by a demographic group. Beyond the results presented here, the NetLogo tool allows individuals to explore what happens to career success as the level and type of (dis)advantage given to one group over another varies.

Our work follows previous demonstrations of how “minor” biases impact future success, especially that of Martell and colleagues [3] who simulated the cumulative effect of a small disadvantage for women in the promotion process of a hypothetical company. In addition, our goal of understanding the accumulation of relative advantage/disadvantage between demographic groups can be situated within the much broader field of inquiry into the dynamics of success [25–27]. Merton [28] introduced the idea of the “Matthew effect” to describe the phenomenon where scientists of high status are given disproportionate rewards for their work compared to less well-known individuals. Recent randomized experiments [29] and empirical studies related to grant funding [30, 31] continue to find evidence, with interesting caveats, that status is an important component of how achievements are recognized and rewarded. Other studies have found that achievement alone (such as publications) is enough to explain some disparities in grant-funding [32] although cumulative advantage still plays a role in the number and quality of publications. Our work is distinguished within the literature by the fact that, like Martell [3], the group identities we use to define relative advantage are constant in time. That is, they are not determined by a quantity (income, success, etc.) that may be subject to the Matthew effect. Both advantaged and disadvantaged populations in our model will experience “success-breeds-success” dynamics, but they will experience it differently: we draw from the social science literature to justify the introduction of a systematic bias in the parameters describing the experience of the disadvantaged population, and study the compounding effects over time. This “group identity” version of cumulative advantage is closer in spirit to Blau and Duncan’s early analysis [33] of occupational data to show that Black men suffered from a cumulative disadvantage compared to White men. For a review of cumulative advantage models and applications, see [27].

We introduce the components of the framework and a particular model within the framework in the next two sections. Then, we present literature identifying disparities between demographic groups and demonstrate the effect of including such disparities in our model. We show how model elements can be adjusted to reproduce observed data. We conclude by positing that this modeling tool serves as a practical demonstration that could be used to engage STEM faculty in productive conversations about accumulative advantage and disadvantage in the career advancement of faculty. Our work joins other recent literature bringing an analytic modeling approach to understanding the role and effects of bias in disparate outcomes among social groups in academia [34, 35].

## RASE: A framework for modeling an academic career

**Fig 1:**
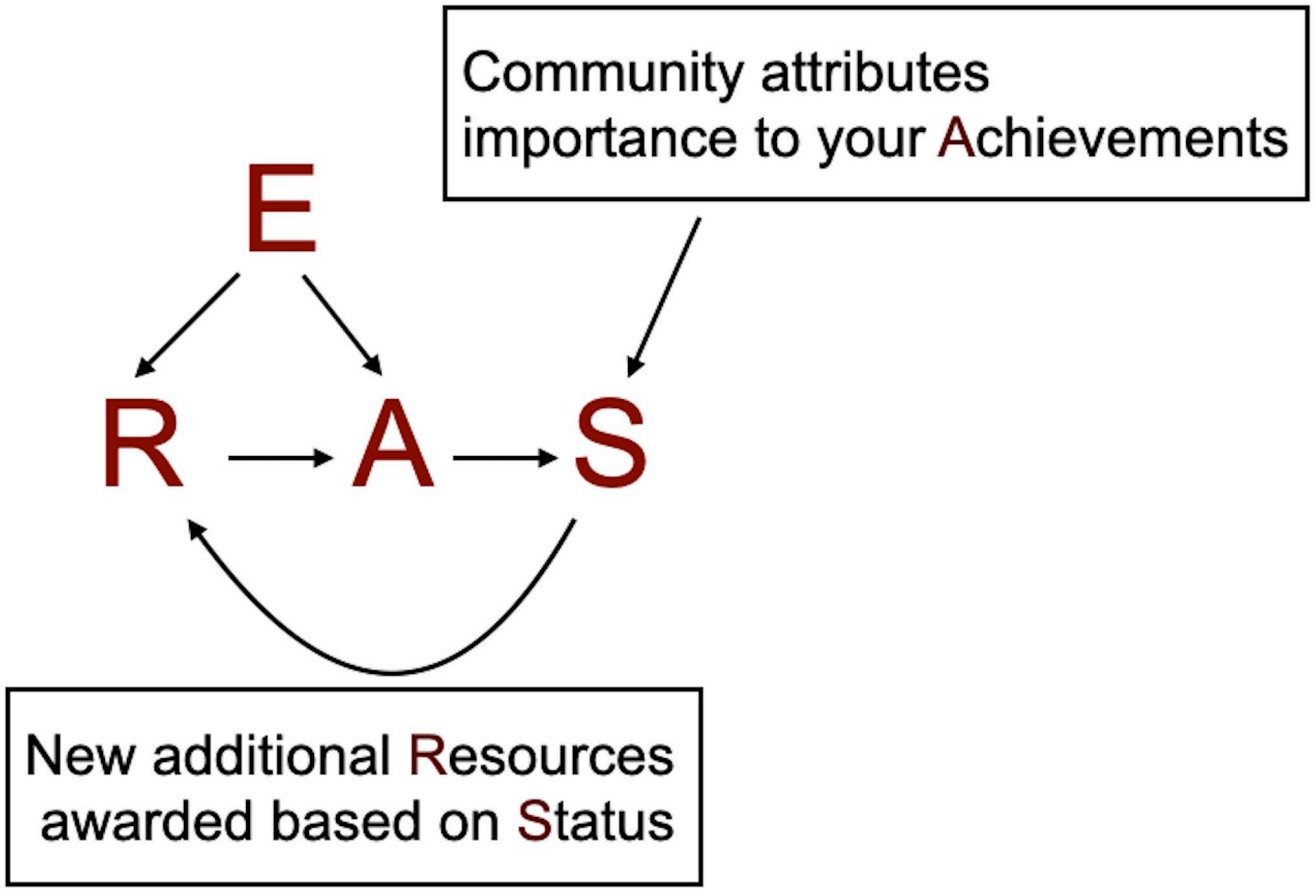
The RASE cycle, where R stands for Resources, A for Achievement, S for Status, and E for Events. Resources are used to accrue Achievements. The scientific community accords Status to your Achievements. New additional Resources are then given based on Status, and the cycle repeats. At each time step, there is a probability to experience Events that impact Resources or Achievements.

In a coarse-grained view, academics use their time, knowledge, and network of collaborators and personnel to produce new knowledge and understanding. If the community judges these contributions to be useful, the academic is rewarded with additional resources and prestige which the individual uses to further advance their work. We therefore propose a modeling framework where at each time step an individual uses all their available Resources (R) to generate Achievement (A) according to a specified function. The individual is awarded Status (S) by the community for their Achievement, where as Merton noted [28], factors other than the Achievement itself may affect the Status awarded. Additional Resources are then allocated for the next time step as a function of Status and the cycle repeats. At each step, individuals have some probability that their Achievement and/or Resources are affected by an isolated Event (E) (Fig 1). In a meritocratic world, each individual would have Status identical to their Achievement and would be equally likely to encounter Events of statistically equivalent impact.

In the context of an academic career, we define *Resources* as things that allow individuals to perform their work, including personal characteristics (knowledge, technical skills, creativity, focus), monetary resources (grants, start-up funding), social resources (time, professional networks, social support), and personnel (graduate students, postdocs). Our framework’s implicit valuation of such incommensurable items is a necessary simplification of reality that mathematical modeling entails. Some Resources, such as knowledge and skills, are always accessible to an individual over the course of their career. Others, such as grant money or graduate students, have a finite lifespan of a few years. Although resources of both types accrue over the course of a career, we make a simplifying assumption that early-career Resources are permanent, whereas those accumulated later have the same, finite lifetime. One way to model differences among individuals within a group is to allow their starting Resources to vary; this is what we will do in the analysis presented here. Some specific types of resources, especially grant funding and laboratory access, may be much more important to workers in some fields than in others (for example, an immunologist compared to a mathematician). On the other hand, time and the mental and physical space to focus on research are universal necessities; our broad conception of Resources allows our framework to capture differences in the availability of such space as experienced by different groups.

*Achievements* consist of anything one has put effort into and would list on a CV, including publications, some awards, invited seminars, students mentored, promotions, and leadership positions in scientific collaborations, societies, or universities. Some elements in an actual career may affect both *Resources* and *Achievements*, such as a grant that is particularly prestigious or an award that comes with money. Other awards might be most properly classified as Status indicators. Differences between individuals could be modeled by allowing variation in the rate at which they convert Resources to Achievements, although we will not present results using this choice here. As with Resources, the typical number and type of Achievements will vary by discipline and sub-field. One of the most universal Achievements is the publication, but publication rates differ substantially across disciplines and sub-disciplines [36]. While there have been attempts to use measures of publication productivity and impact, for example the h-index [37], these attempts fail to provide measures comparable across fields. Additionally, key gate-keeping aspects of academia, especially promotion and tenure, do not proceed purely according to publication rate. For these reasons, we choose a broader definition of Achievement here.

*Status* is signaled primarily through credit or accolades bestowed by others. This may include awards, invitations to high-profile events or speaking positions, tenure, promotion and named professorships, and honorary positions or affiliations. Since Status is subjective, it is best defined as an inferred parameter. This is how we use it in our data comparison, where we interpret a colloquium invitation at a high-profile institution as an indicator that an individual has passed a certain threshold in Status. We use the fraction of the population receiving an invitation to determine the value of the Status threshold in the model.

For the quantitative implementation of the model we detail below, we have chosen ballpark numbers most appropriate for biology, chemistry, or physics, although even within those disciplines there is significant variation by sub-field. To truly calibrate the numerical values appropriate for the baseline model would require longitudinal studies of multi-dimensional Achievements. Few such studies exist, and those that do are generally designed to examine a specific outcome and so choose a specific, restricted population to study (for example, [38]). However, our primary goal is to demonstrate how small differences between demographic groups can compound to generate significant differences in career outcomes. We anticipate that future studies will allow the model to be refined.

We chose to run the simulation where Achievement, Status, and new Resources are calculated in six month intervals. (This choice is arbitrary and does not affect the outcome.) Our initial choices of parameters and numbers are chosen so that 5 units of Resources are required to produce 1 Achievement, which roughly corresponds to one typical-impact publication. To apply the model to a field with a lower publication rate, one can re-interpret the units so that each typical publication generates, say, 2 or 3 units of Achievement. In a typical simulation without Events, the modal number of achievements in the last year of a 40- year career was around 25. While our scale is set such that we expect one unit achievement to be one typical impact publication, achievement is meant to measure more than just publications (e.g., it also may reflect leadership positions, grants, and presentations).

We model differences among individuals by drawing initial Resources *R*(*t*_0_) from a normal distribution with mean 4 and standard deviation 1. This choice corresponds to assuming that most beginning students do not quite have the resources to produce a publication, although some may. Formally, for each time step *t*_*i*_, Achievement is given by *A*(*t_i_*) = *aR*_Total_(*t*_*i*−1_). The parameter *a* is chosen to be *a* = 0.2 to give the 5 to 1 ratio between Resources and Achievement. Status is accorded via *S*(*t*_*i*_) = *A*(*t*_*i*_) (assuming a meritocracy) and new resources are awarded via *R*_new_(*t_i_*) = *rS*(*t_i_*) where we take *r* = 0.4. This number was set to provide a reasonable growth and overall scale of Resources and Achievement. We treat early career Resources as permanent and subsequent Resources as having a finite lifespan. To be specific, we choose Resources to accumulate as permanent until 8 years into the career, after which time they contribute a fixed additional amount for 5 years. The transition at 8 years corresponds approximately to the transition to junior faculty, while the 5-year window is an approximate time scale over which start-up and grants might persist. In other words, up until year 8, *R*_Total_(*t*_*i*_) is the sum of all resources accumulated. After year 8, *R*_Total_(*t*_*i*_) is the total accumulated up to year 8, plus any resources gained in the 5 years prior to time *t*_*i*_.

Due to the linear functions we employ in our model to relate R, A, and S, Achievement in the early career phase grows exponentially (as is familiar from compound interest). In later years, as the finite-lifetime Resources come to dominate, Achievement plateaus. In a model that is deterministic except for the initial normally distributed allocation of Resources, *R*(*t*_0_), the distribution of Achievement after 40 years is also a normal distribution, and the individual who started with the most Resources finishes with the highest Achievement. In addition, the shape of each career trajectory is the same. Neither of these features, which clearly conflict with reality, will remain once the stochastic Events are added.

Our basic model choices, prior to adding stochastic Events, might be modified in various ways. As described above, our model allocates Resources at each time step in proportion to Status, in line with the intra-individual success-breeds-success dynamics of Shea and Crystal [39] recently modeled in [40]. One could add a Merton-style Matthew effect by allowing the updated Status at each time step to be a function of both prior Status and new Achievement, similar to the early work of de Solla Price [41] and Rosen [42]. In addition, one might posit that the parameter *a* should be drawn from a probability distribution to account for individuals differing in their capacity to convert Resources into Achievements.

Such modifications would entail a trade-off between accuracy and simplicity; here, we tend to opt for the latter, recognizing that no matter how complicated the model, it serves only to illustrate the potential long-term effects of small differences compounded over time rather than to capture reality with perfection.

### Stochastic events

Many important events that influence a career trajectory are not within the individual’s sole control, but are instead related to the professional or personal environment. Individuals may speak about the most significant of these events as “being in the right place at the right time” or as good or bad “luck.” For example, Events that positively impact an individual’s Resources might include being asked to join a grant proposal initiated by others, or finding low-cost, convenient childcare. Being assigned a particularly heavy committee load or having a family member with a serious illness would be negative Resource Events. Achievement could be enhanced by being asked to join an experiment or paper initiated by others, or sparking a brilliant conference submission idea over dinner with colleagues. Negative impacts on Achievement come from Events such as receiving a particularly unfriendly referee report from a prestigious journal or not being nominated for an award despite being qualified.

Though some may feel that randomness plays no part in a successful career—that success is determined completely by pluck rather than luck, as it were—that narrative has been challenged [43–45]. There are undeniably many circumstantial factors influencing an academic career that are difficult to model precisely. This is the purview of the statistical paradigm, in which variation is described using probabilistic models.

**Fig 2:**
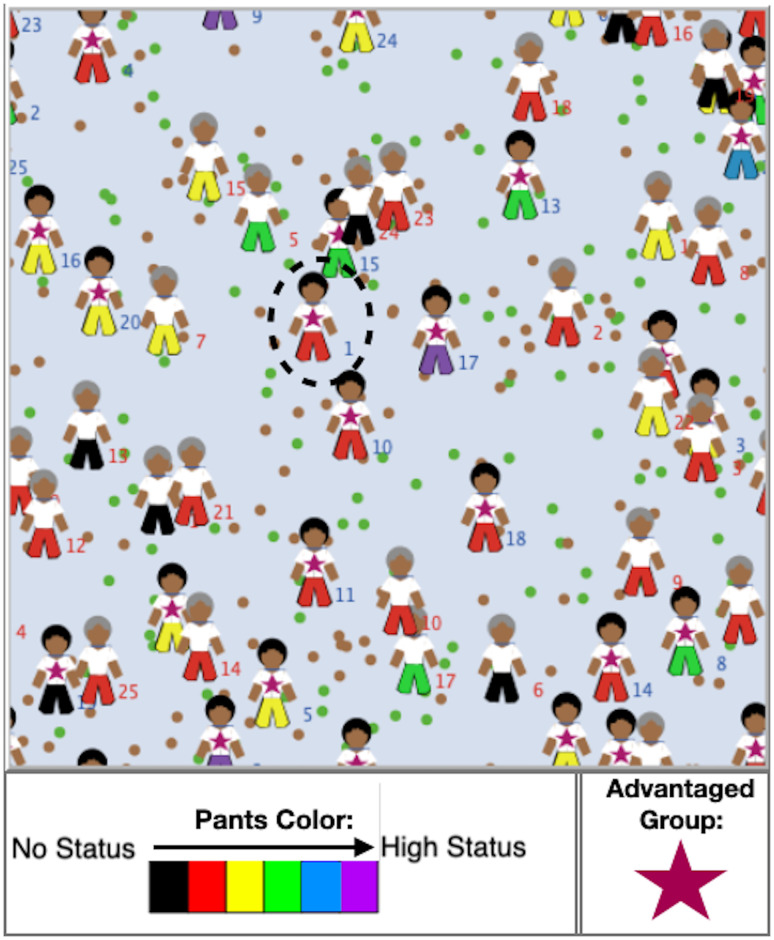
A screenshot of NetLogo’s graphical interface illustrating career outcomes in our model. Advantaged individuals have stars on their shirts, and the pants color of all figures updates at each time step to indicate their Status. The numerical label next to each individual is their position in their population’s starting Resource distribution (1 had the most initial Resources). Resource (Achievement) Events experienced by both populations are represented by brown (green) dots. A large number of extra Events affecting only the disadvantaged population are not shown to simplify the image. Notice that no disadvantaged people (shirts without stars) have status pants above the green level, and that the individuals that started with the highest Resource level (e.g., circled figure) need not end up with the highest status level after 40 years: the stochastic Events significantly affect eventual success.

To model and visualize the impact of stochastic environmental Events, we use NetLogo [24] to place hypothetical academic “people” on a two-dimensional (wrapped) landscape containing a population of Events that can instantaneously alter either Resources or Achievement (Fig 2). The Events and people have random starting positions. Events move one unit (about 1/30th of the size of the landscape) in a random direction at each time step, while people remain at their original location. The two-dimensional landscape allows for an easy visualization, and the choices about motion and placement of people and Events are only relevant in that they assure that each individual, on average, encounters one Event (affecting either Resources or Achievement) per year.

When an individual encounters an Event affecting Resources (Achievement), its magnitude is drawn from a normal distribution with mean 5 (1) and standard deviation 2.5 (0.5). Then, the sign of the event, positive or negative, is chosen by a coin flip. We enforce Achievement ≥ 0 by replacing any negative Achievement values by 0 at each time step. In some models of the Matthew effect (e.g., de Solla Price [41]), the distribution of stochastic Events is a function of prior success. Here, we consider Events that are always drawn from the same distribution, and “success breeds success” compounding occurs due to our deterministic cycle of Resources-Achievement-Status.

**Fig 3:**
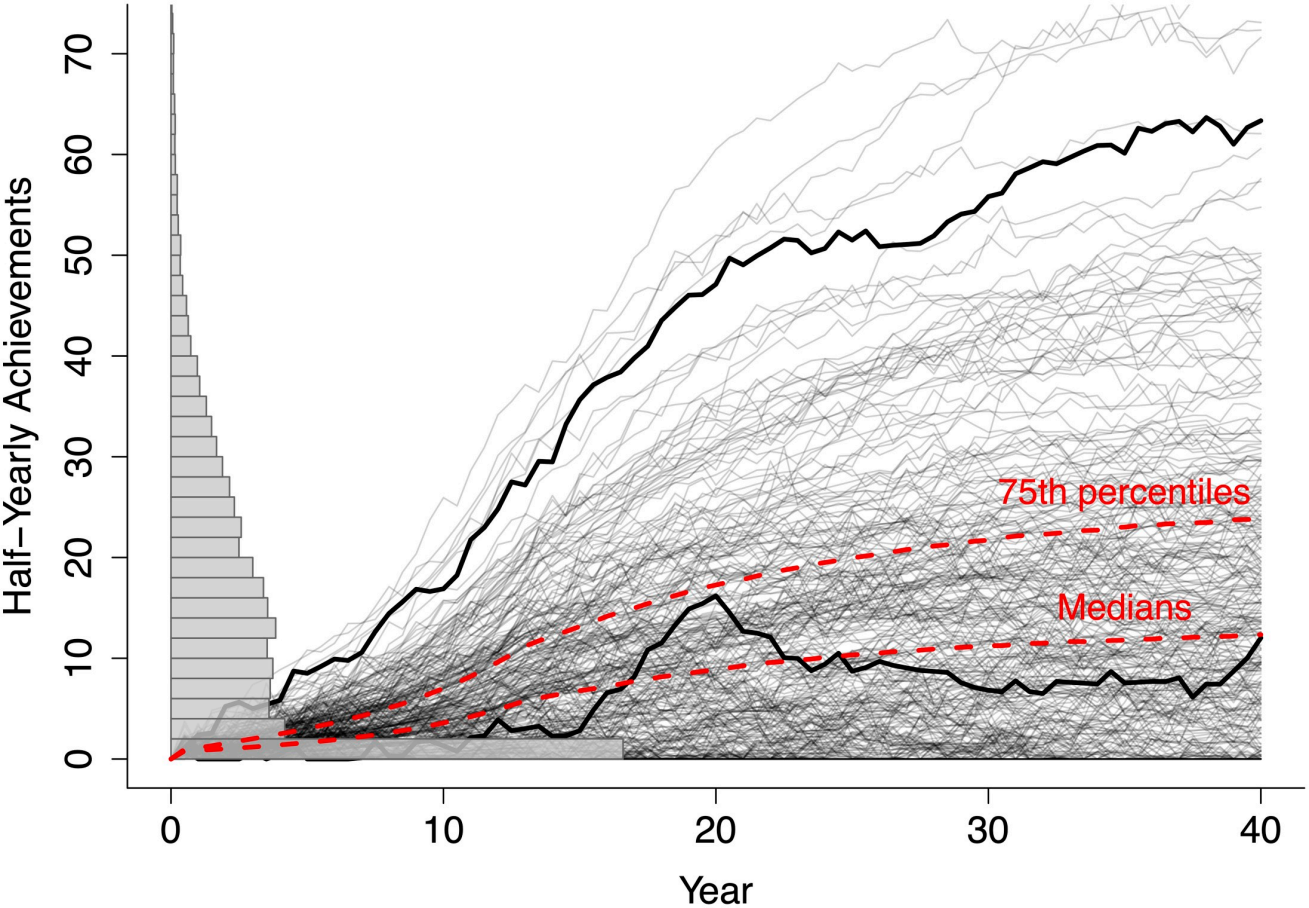
300 sampled career trajectories of achievements over time, *A*(*t*), are shown with a histogram of final-year values for all 20,000 simulated individuals. The histogram reveals a large number of final-year zeros. Two highlighted trajectories show in one case that early positive results often produce career-long advantages even in the face of random buffeting by positive and negative events; in another case, a slow start makes even a surge of fortunate events difficult to capitalize on. The dotted red lines depict the trajectories of the yearly medians and 75th percentiles.

Fig 3 shows how Achievement accrues within this model for the RASE cycle. One important effect of adding the randomly encountered Events is that starting Resources no longer determine final Achievement; that is, trajectories are no longer monotonic and they may cross. Trajectories may show bursts of activity, spurred by and perhaps ended by external events, similar to hot streaks [46].

A second effect of the Events is that they generically concentrate final Achievement into the hands of fewer individuals by the end of the 40-year period. Pluchino et al [47] have previously suggested that a distribution of wealth or status following the Pareto Principle, with 20% of individuals controlling 80% of the wealth, can be attributed entirely to “lucky” events [47], and wondered if a similar law holds in academic careers. In our model, we find that Status is not accumulated so extremely by just a few individuals. Analyzing all individuals after 40 years, we find that for the parameter choices used above, the top 20% of people have about 51% (rather than 80%) of the total status. Interestingly, we find that some simple changes, which may more accurately reflect some aspects of reality, can increase the level of inequality. For example, if the Events are labeled as positive or negative before the simulation runs, then some individuals will happen to be placed in environments with more positive (or negative) Events than the mean. The variance in the net effect of the Events goes up, and so does inequality. Of course, we might alternatively apply a status multiplier as suggested by early literature on the Matthew effect and look at the distribution of Status rather than Achievement. Since Status can be inflated in a way that Achievement, which requires actual effort by an individual, cannot be, Status is more likely to display the more extreme inequality of a Pareto distribution with a power law close to 1.

## Evidence of differential career outcomes

While the model above can be applied to any academic career, our main interest here is to use it to understand how trajectories of different demographic groups might diverge. Though *all* faculty members encounter instances of advantages and disadvantages across their careers, we consider the consequences of having a small but systematic assignment of group-based advantage and disadvantage that can spur or inhibit academic progress, respectively [1, 5]. For readers unfamiliar with research on academic advantage and disadvantage, we provide a broad review to highlight the extensive literature on academic disparities and how they operate through the RASE framework. We aim to paint a general picture of the evidence, though we recognize the reviewed findings are complex and conditional. Not every member of an underrepresented group will be affected by all possible disparities, but this literature in the aggregate provides a basis for varying Resources, awarded Status, or statistics of the Events experienced by different populations. We use the term “underrepresented” to refer to groups numerically underrepresented as STEM faculty and underrepresented in the pool of STEM graduates [48]. We thus refer to White men and Asian men as “majority groups” and White women and other racial minorities as “underrepresented groups,” though underrepresented status may also constitute disadvantage by sexual orientation, nationality, ability status, and socioeconomic background. Given that challenges faced by individuals in STEM are compounded by having multiple marginalized identities (e.g., women of color; [14, 19]), our model may be applied to any of these cases by adjusting the differences between two (or more) modeled populations.

### Resources

To excel in academia, faculty must acquire resources that facilitate their achievements and career status. Resources include personal characteristics (e.g., training, skillsets), financial status (e.g., funding), and interpersonal and structural supports (e.g., protected time, professional networks, graduate students, personal relationships) that could engender future success. However, the dispersion of resources across faculty groups is neither consistent nor equitable.

Funding for research lays a foundation for productivity and subsequent financial support. In some disciplines, women receive less start-up funding than men [49], and underrepresented groups in the Netherlands, the U.S., and Canada receive fewer and smaller grants than their majority counterparts, while controlling for factors such as productivity and institutional prestige [15–18, 21]. Among recipients of career development awards from the National Institutes of Health, women report having less adequate grant support than men [50], suggesting that some groups are disadvantaged both in the amount of funding and in procuring support for translating funding resources into potential achievements. At the interpersonal level, underrepresented groups tend to be less connected to high-status contacts who promote and mentor them through the academic pipeline [51, 52]. White male prospective students, for example, are more likely to receive a reply from faculty when they seek to discuss research [53], and male faculty are more likely than female faculty to receive a response from other male faculty when seeking research materials [54]. Men’s greater access to other men may provide lucrative opportunities; as one example, an analysis of publication history reveals that co-authoring with high-status collaborators early in one’s career provides downstream advantages to one’s career trajectory [12]. If men tend to be the high-status collaborators in some fields and are more likely to be connected to other men, it stands to reason that junior women may be at a disadvantage in this respect.

Having time to dedicate to research also fosters productivity, yet time may be differently available to faculty. Underrepresented groups’ greater responsibilities with service [55], mentoring and teaching [56], and accommodating student requests [57], and, in some cases, contending with an inequitable gendered division of labor in the private sphere [58] constrains time available for research. Likewise, underrepresented groups may experience strain on cognitive resources. Experiences of stereotype threat, for example, can burden cognitive resources and work performance through physiological stress responses, working memory capacity, and self-regulation efforts [59]. People of color endure what some scholars describe as an “inclusion tax,” or the additional resources required (e.g., time, emotional energy) to navigate norms in predominantly White academic institutions [60].

Though all faculty may experience unprecedented obstacles (e.g., death in the family; illness; the COVID-19 pandemic), some encounter more severe challenges because of their gender or race. For example, some faculty contend with negative, unwanted experiences like sexism and racism that deteriorate their psychological health (e.g., microaggressions, harassment). Nearly two thirds of women conducting scientific field research reported they had experienced harassment and assault [61]; more broadly, national reports identify academia as a primary site for sexual harassment, which is detrimental to women’s personal and professional well-being [62]. Consequently, some have noted a “harassment tax” suggesting harassment drains one’s emotional, psychological, and practical resources (e.g., through reporting procedures) and thus taxes productivity potential [63].

We emphasize that resources represent a broad set of supplies. For example, grants may be highly-valued resources by academics in some fields (e.g., STEM, social sciences), whereas those in the arts and humanities may seek fellowships and travel opportunities as resources to support their productivity. Further, across disciplines, some resources are likely appreciated by all, such as good training, time, freedom from distractions, and support for one’s commitment to their scholarship. Resources are thus relevant to all those who are seeking a solid foundation for an academic career.

### Achievement

Disparities in achievement emerge as an artifact of resource allocation and bias in the achievement-earning process. Some resources (e.g., large-scale funding, access to social networks) generate achievements; further, when faculty are recognized for resources and achievements, they may receive the opportunity to collect more achievements. However, not all opportunities for achievements are created equal. Some research on the review process suggests that bias in some disciplines may impede achievement. A few studies document that double-blind systems, compared to when authors’ identities were known, yield more accepted publications and conference abstracts for women and scholars who lack fame or institutional prestige [64–66]. A similar pattern is detected in the domain of teaching, in which racism and sexism influence evaluation [67, 68]—when women’s identities are known, they receive less favorable evaluations. For example, in an experimental paradigm, students rated an online instructor who operated the class with a male alias more positively (e.g., as more prompt and fair) than an instructor who presented as a woman, regardless of the instructor’s actual gender and despite synchronizing the timing of instructor feedback across conditions [69]. Finally, the accrual of achievements may be boosted or impeded by various events. Even when encountering the same unexpected event, such as the COVID-19 pandemic, the impact on achievement outcomes may be different. There is preliminary evidence of disparities in pandemic-related hardships on potential achievements, such that career setbacks during the pandemic are disproportionately endured by women and racial minorities [70–72]. Indeed, the accumulation of achievements is subject to extrinsic factors.

### Status

In light of meritocratic values in the United States, people may be motivated to believe that status is purely a function of achievement in STEM [73]. However, though resources and achievements can promote status, underrepresented groups’ resources and achievements may be converted into status at a lesser rate than that of majority groups. Compared to women, men receive greater recognition for their contributions in collaborations [74, 75] and more glowing accolades [22, 76] and less doubtful endorsements [20] in letters of recommendation, such as being referred to as “brilliant,” “genius,” and “trailblazing.” Publications with female authors are less frequently cited by male-led teams [77]. When members of underrepresented and majority groups hold identical records of achievements (e.g., GPA, productivity, skillsets), evaluators rate underrepresented group members and their achievements as less worthy of hiring and compensating, as found in multiple CV audit studies [19, 78, 79]. Beyond the entry point of hiring, majority groups are also more likely to hold distinguished or named faculty positions [80] and to receive scholarly awards for career success [81]. Across career milestones—from laboratory managers [78] to postdoc applicants [76] to candidates for full professor [6]—underrepresented groups with comparable achievements as majority groups tend to be at a relative disadvantage.

We acknowledge that our review of resources, achievements, and status simplifies their interconnected nature, and we caution readers from defining these components as discrete categories. Indeed, faculty who have acquired status, in turn, may use that status as a resource toward gaining achievements, which can create a cyclical and complex process of attaining resources, achievements, and status. This process may be particularly characteristic of academia given that the established road to success depends on the resources and achievements one accumulates across an academic lifespan.

## Model applications

The RASE framework can be used to investigate “in theory” the impact of disparities on academic careers or it can be used as a tool to model empirically measured inequities. In this section we illustrate both of these applications. First, we show how unequal representation develops among high-status individuals, as predicted by our model when several disadvantages documented in the literature are included: unequal initial Resources, differently awarded community Status, and microaggressions—a disparity in experienced Events. In this scenario, the disadvantaged group receives initial Resources 5% lower, on average, than those of the advantaged group: The mean of the disadvantaged group is 3.8 while the mean of the advantaged group is 4. The Status awarded to the disadvantaged group per Achievement is 95% of the Status awarded to the advantaged group, i.e., *S*_Disadv_(*t_i_*) = 0.95*A*_Disadv_(*t_i_*). Finally, the disadvantaged group is subject to an extra population of negative Achievement Events designed to represent the effect of microaggressions and low-level harassment due to race or gender. A typical disadvantaged individual will encounter 10 extra Events each year, with magnitude drawn from a distribution of mean 0.03. In other words, the Events on average cost a disadvantaged individual 30% of one Achievement (e.g., 30% of a typical impact publication) each year. Keep in mind (cf. Fig 3) that 20 Achievements per year is typical in the second half of a career without any disadvantage in this model.

**Fig 4:**
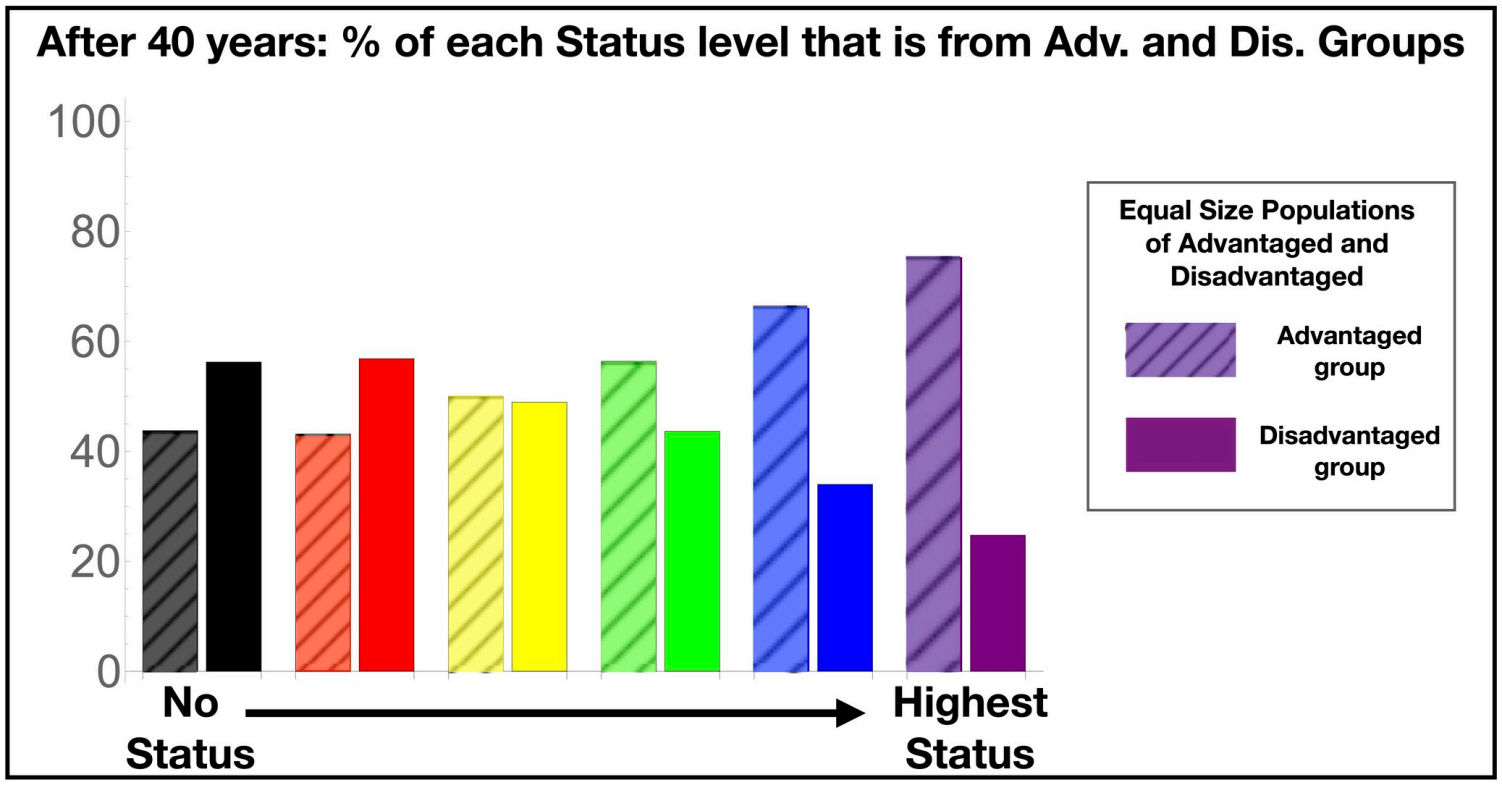
The cumulative effect of disadvantage over a 40-year career affects the percentages of disadvantaged members at each status level, assuming equal numbers in the advantaged and disadvantaged populations. The disadvantaged population began with 5% fewer resources on average, was awarded 5% less status per Achievement, and on average experienced 10 low-impact negative Achievement events each year. After 40 years, the representation of the disadvantaged group among high status individuals falls to about a third. For reference, the highest two status categories together contain about 10% of the individuals.

Fig 4 shows the results for several equally-spaced Status levels: After 40 years of statistical disadvantage at the few percent level, the representation of the disadvantaged group in the highest two Status levels (roughly the most successful 10%) falls from parity to about 1/3. This scenario was also used to generate Fig 2 for a small number of people, but with statistics still representative of those shown in Fig 4.

Our framework can also be applied to model observed disparities in academia. As a specific example we look at the analysis of Nittrouer and colleagues [82] who found that men were more likely than women to be colloquium speakers at prestigious universities after controlling for gender and rank of available speakers.

For academics, invitations to present at colloquia relate to each component of our model. They are Achievements that one lists on a CV, Resources that can establish personal connections and provide opportunities to discuss new ideas, and Status enhancers due to the exposure one’s work receives as a result. They can also cause, or be caused by, serendipitous Events that are difficult to predict simply based on one’s cumulative Resources, Achievements, or Status. Here, we apply our model to explore how a variety of factors could create the gender disparity observed by Nittrouer and colleagues [82]. For simplicity, we will focus only on data for assistant professors in two of the six fields studied, biology and history. We assume that faculty members at this stage are in year ten, assuming an academic career begins at the start of graduate school. Thus, using our 6-month time step, we consider individuals’ cumulative Status at the end of time step 20.

The faculty members who were invited to speak at colloquia are assumed to be those who surpassed a particular threshold of cumulative Status, that is, Status values summed from time = 0 to time = 20 in our simulations. We calculate this threshold as the top fraction that matches the fraction of invitees observed by [82]. For instance, the biologists comprised 1,134 assistant professors (470 female and 664 male), of whom 289 (101 female and 188 male) were invited speakers. In this case, our threshold is the top 289/1,134, or 25.5%, of simulated cumulative Status scores. A similar thresholding analysis could be used to model the tenure and promotion process, or the “leaky pipeline” problem, if individuals below a certain Status level are dropped from the population.

We will consider several ways to model disadvantage in turn, determining in each case the magnitude of disparity required to achieve the level of inequity shown in the data. First, we assign different means to the Gaussian distributions of initial Resources assigned at time = 0, keeping all other model parameters at parity. We find that assigning the disadvantaged group initial Resources from a distribution with a mean of 70% that of the advantaged group (2.8, rather than 4, in our units) explains the data. To contextualize this number, consider that in the Unites States, the income of ethnic minority groups in an average metropolitan area, especially Black Americans, is indeed 30-40% below the average income of non-Hispanic Whites [83].

**Fig 5:**
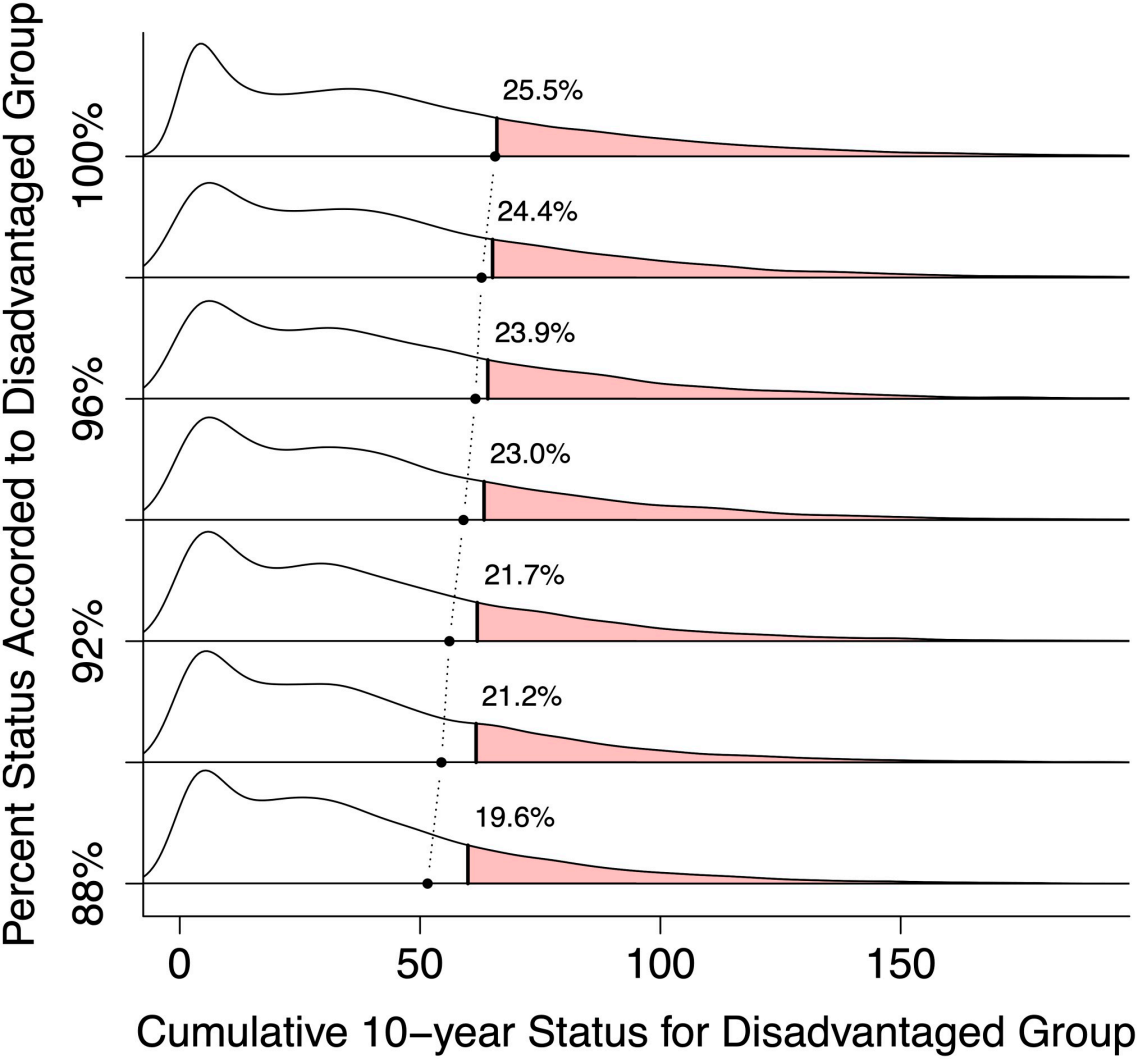
The shaded portions of the density plots depict the individuals who exceed the threshold for being invited to present a colloquium in our biology faculty example. The threshold is the top 25.5% of a combined population consisting of 41.4%, or 470/1,334, disadvantaged individuals (see Table 1). The topmost density corresponds to no relative disadvantage. Moving down, we see that lowering the Achievement-to-Status multiplier—while keeping all other aspects of the simulation constant—diminishes the percentage exceeding the threshold. The dotted line depicts the cutoff for the upper 25.5% of the disadvantaged group only.

Second, we accord different values to the parameter *s* that determines community-perceived Status based on Achievements via the formula *S*(*t*_*i*_) = *sA*(*t*_*i*_). One group is assigned *s* = 1 (100%) to represent an ideally meritocratic system, whereas the disadvantaged group is assigned a smaller value of *s*. The results of implementing this second idea are depicted in Fig 5, which shows the distributions of 10,000 simulated career trajectories for the disadvantaged group as a function of *s*. At the top where *s* = 1 (100%), we observe no disadvantage at all in the “disadvantaged” group, which attains the same 25.5% above-threshold rate seen in the overall population, because both groups share exactly the same sets of parameter values. As *s* is gradually decreased, we reach a point near *s* = 0.91 (91%) at which the proportion of the disadvantaged group achieving the invitation criterion matches the observed proportion of female colloquium speakers, 21.5% [82]. In our highly idealized model, therefore, we can replicate the observed overall and female-specific rates of colloquium invitations by setting the women’s perceived status as a function of achievement around 10% below that of men’s, *assuming no other group differences*.

Finally, we might assume that the two groups are subject to different stochastic Events. Here, we create negative Events to which only the disadvantaged group is potentially subject, according to two different scenarios. One scenario adds small negative values to one’s Achievement score—we might envision microaggressions that predominantly affect only a particular subgroup, for instance—drawn from a Gaussian distribution whose mean and standard deviation are one tenth that of the other stochastic Achievement-specific Events in our model. Another scenario adds large negative Events, perhaps suggesting harassment or discrimination, using a distribution whose mean and standard deviation are ten times as large. Aside from these additional hits to Achievement, each scenario holds the two groups’ model parameters the same, including the presence of other randomly-occurring Events that can be positive or negative.

Table 1 reports the approximate simulation parameter settings that achieve the same percent of above-the-threshold percentages for the disadvantaged group as observed by [82] for the female group. These settings entail each of the four cases described above. In this application, we have not included any scenarios in which multiple parameter values differ between the two subgroups, nor have we considered other instances (e.g., observed discrepancies between racial groups) in which it is possible to model one group as disadvantaged relative to another based on the Resources, Achievements, and Status that typically drive success in academia.

**Table 1:** Model parameter settings in four simulation scenarios that achieve the observed percentages of women above a threshold of cumulative status observed among assistant professors in two fields as reported by [82]. Counts and percents for men may be obtained by subtraction. The simulations involve two subpopulations for which all settings are identical except the one being varied only for the relatively disadvantaged group. Further details about each of the four scenarios are given in the main text.

Field	Above Threshold		Scenario (see below)			
	Total	Female	(a)	(b)	(c)	(d)
Biology	289/1134	101/470	70%	91%	5.1	0.55
	(25.5%)	(21.5%)				
History	112/626	52/308	90%	97%	2.8	0.32
	(17.9%)	(16.9%)				

(a) Percent of mean initial resources

(b) Percent of Achievement-to-Status multiplier

(c) Mean annual additional small hits to Achievement

(d) Mean annual additional large hits to Achievement

The examples in Table 1 demonstrate that a particular disparate outcome for one group relative to another can be achieved by multiple distinct sets of parameter-value settings. We view this flexibility as a strength of our modeling framework because it encourages consideration of the breadth of potential mechanisms that produce disparate outcomes, mindful that we can never claim that results from any particular set of model parameters using the framework provides “proof” that the outcomes it produces could only have arisen from those settings.

## Conclusions

The RASE framework with its four elements—Resources, Achievement, Status, and Events—is a tool both to organize and to visualize the impact of accumulated disadvantage in career outcomes. We have argued that many observed disparities in the literature can naturally be discussed and organized within this framework. We have demonstrated how a specific model based on this framework can be used to explore the compounding effects of disadvantage due to group status. It is also able to reproduce the observed disparities in colloquium invitations of [82]. These combined functions allow the RASE framework and associated NetLogo visualization to provide a powerful educational tool.

Diversity, equity, and inclusion trainings are increasingly common in academic units, despite there being mixed evidence regarding their effectiveness [84]. As a result, there is a critical need to identify and test new strategies for effectively teaching about bias in academia. Intervention research suggests that experiential learning interventions, such as being tasked to engage with simulations of the accumulation of advantage and disadvantage, successfully promote knowledge about gender equity and reduce sexist beliefs compared to receiving information without the active learning component [85–87].

Facilitators of active learning sessions, specifically those who work with STEM faculty, can manipulate the RASE tool to demonstrate long-term impacts of disparities and interpret the resulting effects on career success. For example, we have introduced the RASE framework in diversity and inclusion workshops in a College of Science at our large research university. As one activity in the workshop, faculty participants are assigned to a specific framework element (R, A, S, or E) and tasked with brainstorming real-world examples of the element. This activity has elicited a rich and lively discussion of the accumulation of disadvantage and advantage in career success. As an additional benefit to exposing STEM faculty to the RASE tool, people who feel they have experienced systematic disadvantage in their careers based of their group status may feel validated as they see themselves and their lived experiences reflected in this framework. That is, the RASE conceptual framework explicates an individual’s account of how Resources, Achievement, Status, and Events characterize their career trajectory, and the framework lends new language to participants to make sense of their experiences.

There are many avenues for future work, both exploring how differences in the mathematical implementation—for example, of the Matthew effect—manifest in outcomes and using additional data to calibrate and refine the model. For example, in this paper we do not explicitly model the placement of individuals in a network [88, 89]. But we have purposefully built the implementation of the model within a numerical tool, NetLogo, designed to handle networks [24] to allow for future work to extend in that direction.

It would be informative to compare the RASE framework and model to longitudinal studies and large datasets on scholarly inputs/outputs [90] and other datasets where there is some evidence that cumulative advantage plays a role. For example, [32] found that differing publication rates and impact factors could explain differences in NIH funding between Black and White scientists and suggested that a cumulative advantage model could be applied.

Finally, testing the short- and long-term effects of exposure to this framework would be a promising area of future inquiry. We imagine those who interact with this framework, especially in STEM, may appreciate its technical rigor. We encourage future researchers to assess whether this framework can facilitate more critical and careful assessments of people’s academic portfolios through an enhanced understanding of the roles that advantage and disadvantage play in career success.
